# Canine Distemper Virus in Tigers *(Panthera tigris)* and Leopards *(P. pardus)* in Nepal

**DOI:** 10.3390/pathogens12020203

**Published:** 2023-01-28

**Authors:** Jessica Bodgener, Amir Sadaula, Parbat Jung Thapa, Bhijay Kumar Shrestha, Kamal Prasad Gairhe, Suraj Subedi, Kiran Raj Rijal, Purushotam Pandey, Janardan Dev Joshi, Prakriti Kandel, Babu Ram Lamichane, Chiranjibi Prasad Pokheral, Naresh Subedi, Ram Chandra Kandel, Himal Luitel, Navapon Techakriengkrai, Martin Gilbert

**Affiliations:** 1 Cornell Wildlife Health Center, College of Veterinary Medicine, Cornell University, Ithaca, NY 14853, USA; 2 Durrell Institute of Conservation and Ecology, School of Anthropology and Conservation, University of Kent, Canterbury CT2 7NZ, UK; 3 National Trust for Nature Conservation, Kathmandu 44600, Nepal; 4 Department of National Parks and Wildlife Conservation, Kathmandu 44600, Nepal; 5 Central Veterinary Laboratory, Tripureshwor, Kathmandu 44600, Nepal; 6 Directorate of Livestock and Fisheries Development, Province 1, Biratnagar 56613, Nepal; 7 Center for Biotechnology, Agriculture and Forestry University, Rampur 44209, Nepal; 8 Department of Veterinary Microbiology, Faculty of Veterinary Science, Chulalongkorn University, Bangkok 10330, Thailand; 9 Center of Excellence in Diagnosis and Monitoring of Animal Pathogens, Chulalongkorn University, Bangkok 10330, Thailand

**Keywords:** canine distemper virus, CDV, tiger, leopard, *Panthera tigris*, *Panthera pardus*, serum neutralization test, serology

## Abstract

From wild dogs *(Lycaon pictus)* in the Serengeti to tigers *(Panthera tigris altaica*) in the Russian Far East, canine distemper virus (CDV) has been repeatedly identified as a threat to wild carnivores. Between 2020 and 2022, six Indian leopards *(P. pardus fusca*) presented to Nepali authorities with fatal neurological disease, consistent with CDV. Here, we report the findings of a serosurvey of wild felids from Nepal. A total of 48 serum samples were tested, comprising 28 Bengal tigers *(P. t. tigris*) and 20 Indian leopards. Neutralizing antibodies were identified in three tigers and six leopards, equating to seroprevalences of 11% (CI: 2.8–29.3%, *n* = 28) and 30% (CI: 12.8–54.3%, *n* = 20), respectively. More than one-third of seropositive animals were symptomatic, and three died within a week of being sampled. The predation of domestic dogs *(Canis lupus familiaris*) has been posited as a potential route of infection. A comparison of existing diet studies revealed that while leopards in Nepal frequently predate on dogs, tigers do not, potentially supporting this hypothesis. However, further work, including molecular analyses, would be needed to confirm this.

## 1. Introduction

Canine distemper virus (CDV) has been repeatedly identified as a threat to wild carnivores and their conservation [1]. The most dramatic outbreaks have been reported in social animals, such as lions *(Panthera leo*) [2,3], gray wolves *(Canis lupus*) [4] and African wild dogs *(Lycoan pictus*) [5]. In this setting, disease spreads rapidly, sometimes resulting in the collapse of local populations [6,7,8]. However, the extinction of the black footed ferret *(Mustela nigripes*) in the wild indicates that solitary carnivores are no less at risk [9]. Any small, isolated population is vulnerable to stochastic effects, and the additive mortality associated with CDV can have a significant impact [10]. Distemper was first identified as a risk to wild tigers in Russia, where it was found that the presence of CDV could increase the 50-year extinction probability of small populations of Amur tigers *(P. tigris altaica*) by 65% [11]. Since then, the virus has been detected in free-ranging tigers and leopards in Indonesia and India [12,13,14]. Characteristic signs in wild felids have been associated with progressive neurological disease, typically terminating in seizures and death [13,15,16]. Between 2020 and 2022, at least six Indian leopards *(P. pardus fusca*) presented to wildlife authorities in Nepal and died with symptoms consistent with CDV. This retrospective study investigates the seroprevalence of CDV in free-ranging tigers and leopards in Nepal as a first step to understanding the potential impact on these populations. 

CDV, also known as canine morbillivirus, is a highly infectious single-stranded RNA virus in the Paramyxoviridae family. It has a worldwide distribution and can infect a wide variety of carnivore species as well as other mammalian orders including rodents and primates [1]. Transmission typically occurs via direct contact or contact with infectious material [17] and, in some species, morbidity and mortality can reach 95% in naïve populations [18]. Those animals which do recover are strongly immunized. A large population is needed to maintain the virus [19], and threatened taxa are normally infected secondary to spill-over from more abundant hosts [20]. Dogs are the most numerous carnivore on Earth [21], and unvaccinated populations are often presumed to be the source of infection [22,23,24]. However, reservoirs can be complex [25]. In some cases, dogs may just be one contributor among a wider community of hosts [26], whilst in others, they may have little or no role at all [19,27,28].

Disease typically follows the same pattern in all species [14] and is broadly comprised of three phases. The initial phase occurs shortly after exposure and is characterized by transient pyrexia and lymphopenia, as the virus replicates in lymphoid tissue [23]. This is followed by an acute phase, where the virus infects epithelial tissue, resulting in a variety of clinical signs including a crusting discharge from the eyes and nose, respiratory and gastro-intestinal signs, and hyperkeratosis of the foot pads [29]. Some animals succumb at this point, whilst others make a complete recovery. A third group go on to develop neurological disease. This can occur sometime after acute phase symptoms have resolved and will be progressive in nature [24,29]. Many factors can influence the outcome of infection [29], but once an animal enters the neurological phase, mortality is almost inevitable.

Captive felids have exhibited a range of clinical signs [30,31,32], but free-ranging animals typically present in the neurological phase [26,27,32]. Following an initial detection in 2003, CDV has been diagnosed in a further four wild tigers and an Amur leopard *(P. pardus orientalis*) in the Russian Far East [15,16,27,33]. Elsewhere, CDV infections have been confirmed in free-ranging tigers and leopards in Indonesia and India [12,13,14]. Most cases presented with neurological signs including disorientation and a lack of fear. This progressed to seizures and the death of those animals that were not euthanized. Weight loss was also a recurrent feature in cases that were examined clinically, with four out of the five tigers and both leopards being extremely underweight at the time of death [13,15,16]. 

Several methods can be used to investigate canine distemper, and the most appropriate technique will be dictated by the context and samples available. In the acute phase, virus is shed in the feces and various bodily fluids [23] and can be detected using RT-PCR, with sequencing providing insights into genetic lineages and transmission pathways [12,16]. Shedding is generally short-lived and may have ceased by the time animals develop neurological signs. Virus can also be detected in post-mortem samples using RT-PCR or immunohistochemistry [14,34], particularly brain and lymphoid tissues [14]. Serological assays can identify past infections and are particularly useful in free-ranging populations, where animals are rarely handled and active infections may be missed [12,35]. Antibodies are normally detectable 10–20 days after infection [36,37] and typically persist for life [29,38]. Commercial bench top kits for dogs (ELISAs or lateral flow immunoassays) rely on species-specific marker antibodies, normally anti-dog IgG or IgM [39,40] and have not been validated for use in other species. The serum neutralization test (SNT) is the only reliable serological assay currently available for felids.

Tigers are enjoying a resurgence in Nepal, with population estimates almost tripling in the past 12 years [41]. However, globally, the species remains endangered, with between 3726 and 5578 animals thought to exist in the wild (or 2608–3905 mature individuals) [42]. The population is highly fragmented and confined to an area that comprises less than 7% of its former range [42]. Despite significant efforts, poaching and habitat loss remain significant threats [42], and in Nepal, burgeoning infrastructure could see recent gains reversed [43]. By comparison, far less is known about leopards [44]. The species is in decline and is classified as vulnerable by the International Union for the Conservation of Nature, but in many countries, the status is unknown [45]. The current assessment for Nepal dates from 2011 and describes the population as decreasing [46]. This is supported by more recent work which suggests there are ongoing losses associated with poaching, habitat loss and human wildlife conflict [44,47,48]. In both species, the additive mortality associated with CDV could exacerbate other threats [10]. However, leopards may also face a growing risk of exposure. Recovering tiger populations are displacing leopards from national parks and increasing their reliance on domestic prey [49,50]. This increased contact with domestic populations could create new opportunities for pathogen transmission [51]. 

This study used archived serum samples and pseudotype-based serum neutralization assay [52] to measure the seroprevalence of antibodies against CDV in wild tigers and leopards in Nepal. Serological investigations were supported by clinical histories and mortality records. Findings confirm that individuals from both species have been infected, and clinical histories suggest that some of these infections resulted in mortality. Leopards had a higher seroprevalence then tigers, suggesting they may be at increased risk of exposure, but small samples sizes preclude conclusions. 

## 2. Materials and Methods

The study focused on two geographic regions of Nepal, the Terai and the Mid-hills. The lowland Terai runs along the country’s southern border and is home to five national parks: Shuklaphanta National Park (ShNP), Bardia National Park (BNP), Banke National Park (BaNP), Chitwan National Park (CNP) and Parsa National Park (PNP). Between them, these protected areas total 3402 km^2^ and accommodate most of Nepal’s tigers. Inside the national parks, the landscape is a matrix of grassy floodplains and tropical forests, but outside these areas, the region is widely settled and heavily cultivated. The Mid-hills lie north of the Terai and range between 600 and 4800 m. Here, the majority of settlements are found in the valleys, while much of the hillside remain densely forested. The region includes a large portion of Annapurna Conservation Area (ACA). However, unlike the national parks, people are not excluded from living or working in this area. 

The samples analyzed in this study were collected opportunistically and archived in the molecular lab at the Biodiversity Conservation Center, Sauraha, in CNP. They included sera from 28 tigers and 20 leopards. Captures took place between 2011 and 2021 and were carried out jointly by the Department of National Parks and Wildlife Conservation (DNPWC) and the National Trust for Nature Conservation (NTNC). Almost all (47/48) captures were conducted as part of routine wildlife management and conflict resolution. The only exception to this was a single tiger, which was captured as part of a collaring study in 2021. Animals were immobilized using a standard protocol of medetomidine (0.07 mg/kg) and ketamine (3 mg/kg) delivered intramuscularly via dart gun or hand injection. Blood samples were collected during immobilization, and the separated serum was archived at −20 °C.

Most tigers (26/28) were captured in and around the Chitwan–Parsa Complex (CPC; 27°46′ N, 84°53′ E) which includes CNP, PNP and the surrounding buffer zones. The remaining two tigers were captured in or around the Bardia–Banke Complex (BBC; 28°33′ N, 81°65′ E) which includes BNP, BaNP and the surrounding buffers zones. Meanwhile, most leopards (17/20) were captured in the central Mid-hill districts, two were captured in the CPC and one was captured in Koshi Thappu Wildlife Reserve (KTWR; 26°65′ N, 87°00′ E).

Prior to testing, all samples were heat inactivated at 56 °C for 30 min. A modified version of a previously described serum neutralization test [52] was used to screen the heat-inactivated samples. This protocol uses an engineered cell line, HEK293dogSLAM, which expresses the canine signaling lymphocytic activation molecule (SLAM-F1, the receptor used by CDV for cell entry) and a replication deficient vesicular stomatitis virus pseudotype expressing hemagglutinin and fusion surface glycoproteins from the Onderstepoort strain of CDV. The referenced protocol was adapted by substituting green fluorescent protein (GFP) for luciferase as the marker for infection [12]. Cell lines HEK293dogSLAM and HEK293T were supplied by the University of Glasgow. Tests were read by examining wells using an inverted fluorescent microscope under low power. Dilutions which achieved a 90% reduction in the number of infected cells compared to the mean count of four serum-free controls were considered to have effectively neutralized the virus. 

All samples were tested in quadruplicate using a 1:16 serum dilution along with a panel of 20 dog sera as positive controls. Samples which tested positive on the initial assay were re-tested to determine the titer using four-fold serial serum dilutions from 1:16 to 1:16,384. The Spearman–Karber method [53] was then used to calculate the final titer. Samples with a titer of 1:16 or greater were considered positive. Seroprevalence was calculated as the number of animals testing positive, which was divided by the total number of animals tested and expressed with 95% binomial confidence intervals. Population comparisons were conducted using a two-proportions z-test in R [54]. Results were reviewed in conjunction with the clinical histories for the individuals concerned. 

Mortality records from the Central Zoo in Lalitpur and in CNP were reviewed for the period September 2019 to September 2022 to identify additional tigers and leopards that had suffered fatal neurological disease, which was characterized by seizures. 

## 3. Results

### 3.1. Serology Results

In total, nine samples tested positive for neutralizing antibodies to CDV. This included three tigers (seroprevalence 10.7% CI:2.8–29.3%, *n* = 28) and six leopards (seroprevalence 30.0% CI:12.8–54.3%, *n* = 20). The seroprevalences for the two species were not statistically different.

**Figure 1:**
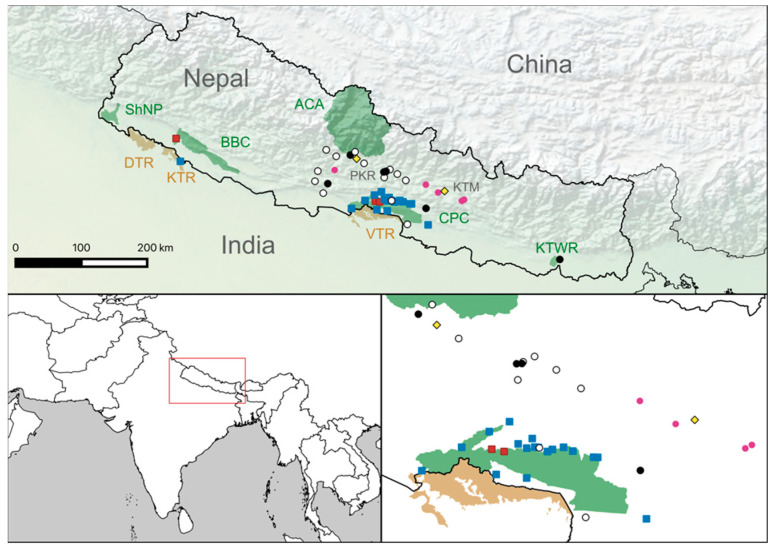
Geographic distribution of samples and results. The locations of tiger and leopard samples are marked by squares (seropositive = red, seronegative = blue) and circles (seropositive = black, seronegative = white), respectively. Unsampled sick leopards are indicated using pink circles. Protected areas are shaded in green (Nepal) and tan (India). In Nepal, these include: Shuklaphanta National Park (ShNP), Bardia–Banke Complex (BBC), Annapurna Conservation Area (ACA), Chitwan–Parsa Complex (CPC) and Koshi Thappu Wildlife Reserve (KTWR). Indian protected areas include: Dudwha Tiger Reserve (DTR), Katarniaghat Tiger Reserve (KTR) and Valmiki Tiger Reserve (VTR). The cities of Kathmandu (KTM) and Pokhara (PKR) are indicated by yellow diamonds.

The three tigers that tested positive all had titers of 128 or less (Table S1). Leopard titers were more variable, with the highest titer reading at 23,168 (Table S2). Seropositive tigers were found in both CNP and BNP, and seropositive leopards were found in both the Terai and the Mid-hill regions (Figure 1).

### 3.2. Clinical Findings for Animals That Tested Positive by SNT

Of the nine animals that tested positive for CDV by SNT, only five were considered clinically healthy. The remaining four exhibited a variety of clinical signs, which are summarized in Table 1.

Tiger Pt12 was a young sub-adult male found outside CNP with injuries to the flank consistent with fighting with another male. During capture, his behavior was reported as being abnormally confident and aggressive. On clinical examination, he was found to be very thin. He was taken into captivity for supportive care, where he refused food. Two days after capture, he was found dead in his enclosure.

Tigers Pt23 and Pt26 both appeared outwardly healthy. Pt23 was an adult female that was captured after attacking a forest guard. She was released inside CNP. Pt26 was an old male in good body condition that had been predating livestock over a period of a month. He has survived in captivity since capture.

Leopard Pp5 was captured in a village and exhibited signs of dyspnea, with open mouth breathing, and tonic–clonic seizures. He was taken to CNP and given supportive care including intravenous fluid therapy (IVFT). Staff reported that he was exceptionally thin and refused food when offered. The seizures progressed, becoming more frequent and severe, and he died the following day.

Leopard Pp7, a young adult male, had the highest titer, at 23,168 (Table S2). He was captured by local people using a net. Veterinary staff noted that he was emaciated, dehydrated and very subdued. Some wounds were present on his head, but these may have been acquired during capture. He was treated with antibiotics, IVFT and supportive care. He was initially reluctant to eat but went on to make a full recovery and was released in CNP.

Leopards Pp8, Pp11 and Pp12 all appeared outwardly healthy and were released back to the wild. Pp8 was found in a chicken coop, and Pp11 and Pp 12 were captured in box traps placed in response to recent conflict events in the area.

**Figure 2:**
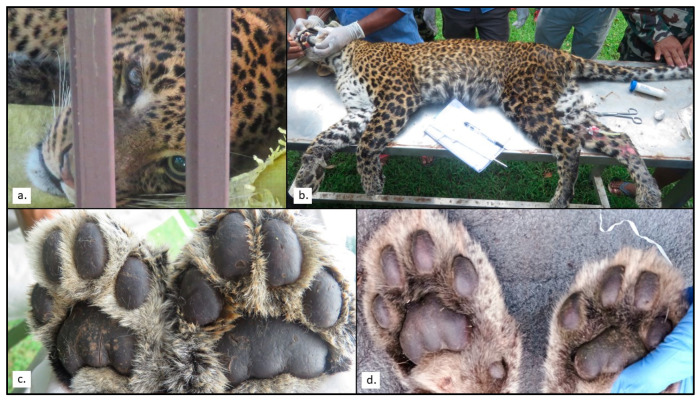
Images illustrating clinical findings for leopard Pp17. (**a**) Prior to anesthetic, in lateral recumbency (prior to drug administration). The left eye (which appears to the right of this image) is markedly enlarged, and the pupil is dilated. The right eye is normal size but appears cloudy. (**b**) In lateral recumbency under general anesthetic. There is poor body condition, which is more pronounced over the hindlimbs where there is accompanying muscle wastage. (**c**) The footpads of Pp17, the skin appears darker and thicker. (**d**) Normal footpads from another leopard, included for comparison.

Leopard Pp17 was captured by the Department of Forest and Soil Conservation and was initially suspected to have fallen from a height. He was emaciated, dull and unresponsive. He was unable to stand and showed signs of hind limb paralysis and muscle wastage. The left eye was swollen, and the left pupil was dilated. The right eye was normal in size, but the eye appeared cloudy. The skin of the foot pads was dark and thickened (Figure 2). He had a poor appetite and died approximately one week after capture.

### 3.3. Mortality Data

A review of clinical histories dating from September 2019 to September 2022 identified six leopards that died with symptoms consistent with CDV (this includes Pp17 and five others where serum had not been collected). These animals are listed in Table S3, and their positions are given in Figure 1. In all cases, animals developed fatal seizures. There were no records of fatal neurological disease in tigers during this period.

## 4. Discussion

Of the 43 carnivore species in Nepal, 21 are threatened with extinction [46]. This study provides the first confirmation that wild carnivores in Nepal have been infected by CDV, a virus that increases the likelihood of extinction of small populations [11,55]. Although the measurement of antibodies is unable to differentiate between infected and recovered individuals, three of the six seropositive leopards had clinical signs consistent with CDV infection, including weight loss, dyspnea and neurological deficits [13,16,32]. Leopard Pp17 also exhibited a characteristic thickening of the foot pads and ocular disease, both of which are well recognized in CDV-infected dogs [17] and were features of the recent case in a Javan leopard (*P. pardus melas*) [13]. Both leopards with neurological disease died (Pp5, Pp17). The only leopard to recover (Pp7) also had the highest antibody titer. The association between high antibody titers and increased survival is well documented in dogs, where convalescent titers can serve as prognostic indicators [56,57]. As such, the circumstances of these cases are highly suggestive of clinical canine distemper infections, although this cannot be confirmed without further testing such as RT-PCR.

Between 2020 and 2022, five other leopards died with acute seizures, consistent with CDV. Unfortunately, serum samples from these animals were not available for testing. Differentials for this presentation include parasitic disease, neoplasia, intoxication, head trauma, rabies, meningitis, metabolic encephalopathies, hypoglycemia and epilepsy [58,59,60,61,62]. Post-mortem examination of future cases and collection of tissues for RT-PCR analysis could confirm CDV involvement and might yield sequence data, potentially providing insights into the identity of reservoir species and transmission pathways [27]. Previously, RT-PCR has been used to detect CDV in a wide range of samples including ante-mortem nasal, conjunctival and rectal swabs and urine [63] and post-mortem tissues, such as tonsil, lymph node, lung, kidney, spleen, brain and skin [64]. The collection of a comprehensive set of samples maximizes the chance of detecting virus [64] and is useful for excluding differentials and identifying any co-infections [2].

A recent paper from India used RT-PCR to identify infections in a wide variety of felids, including tigers and leopards in Uttar Pradesh (UP), which borders Nepal to the south [14]. Amplification and sequencing of partial hemagglutinin (H) genes from two of the tiger samples from UP revealed these isolates were closely related to a dog isolate from the same area, sharing 99% identity. All three isolates clustered with the Asia 3 genetic lineage. Isolates from a lion, a civet cat and another dog, again all from UP, clustered in a separate clade and were associated with the Asia lineage. Meanwhile, amplification and partial sequencing of the well-conserved Nucleocapsid (N) gene revealed isolates from leopards (*n* = 3) and lions (*n* = 3) in UP and neighboring Madhya Pradesh (MP) were clustering together. Interestingly, a palm civet isolate from UP did not cluster with them and instead was more closely related to strains from Germany [14]. All of this suggests there are multiple strains circulating amongst wildlife close to Nepal, and there may even be multiple cycles involving different species. Future isolates from Nepal could be compared to this data and used to investigate transboundary transmission.

CDV readily degrades in the environment; therefore, transmission primarily occurs through direct contact. In social animals, this may occur through grooming, fighting and shared food. Solitary carnivores have low levels of direct contact with conspecifics and therefore reduced opportunities for intra-species transmission. In this context, transmission from other species has increased importance. Predation has been posited as a potential mode of inter-species transmission [27], as it affords the close contact necessary for viral transfer. Infected animals may also be debilitated, making them easier prey. As such, diet comparisons might explain the difference in seroprevalence between tigers and leopards. A review of scat-based diet studies in Nepal published between 2012 and 2022 identified 13 papers that dealt with tigers or leopards [50,65,66,67,68,69,70,71,72,73,74,75,76], of which 11 provided sufficient detail for comparison (Table S4). These illustrate a clear disparity in prey selection, with all studies reporting dogs (or *Canis* spp.) as a component of leopard diet, whereas no dog remains were found in tiger scat. Distemper is known to circulate among dogs in Nepal [77,78] and it is, therefore, possible that higher levels of dog predation could account for the greater levels of exposure in leopards. Competition with Nepal’s growing tiger population [41] has displaced leopards from protected areas and increased their reliance on domestic prey [49,50,79]. If leopards face continued pressure, the consumption of dogs and associated exposure to CDV could increase further. In nearby India, dogs have been found to account for up to 39% of the diet [80,81].

Dog vaccination is only useful as a strategy to mitigate CDV risk for threatened carnivores in areas where they play an integral role in the CDV reservoir [25]. In Nepal, tigers are largely confined to national parks where contact with dogs is limited to areas close to park boundaries and surrounding buffer zones. The authors have seen photographic evidence confirming that tigers in Nepal occasionally do eat dogs, but this occurs far less frequently than in leopards (Table S1). Seropositive tigers were captured close to the boundaries of CNP (<250 m) and BNP (1000 m); therefore, transmission from dogs is plausible. However, in Russia, there was no evidence that dogs were acting as a reservoir for tigers, and instead, wild carnivores were implicated as the most likely source of infection [27]. Elsewhere, dogs appear to be just one contributor to a more complex reservoir community [26]. In these circumstances, vaccination of free-ranging dogs would be unable to prevent infections in tigers [27]. Nepal is home to a diverse array of carnivore species, more than 40% of which have already been identified as susceptible to CDV [1,46]. Further serological and molecular surveys would be required to assess the relative importance of these wildlife hosts in CDV maintenance and transmission to Nepal’s threatened big cats.

In many parts of the world, surveillance for CDV in wildlife is hampered by a lack of access to laboratories equipped with the necessary tests. Access to international laboratories may be impossible due to export bans or slowed by permit application processes (e.g., CITES), creating a barrier to timely surveillance and disincentivizing field researchers. Whilst PCR facilities are widely available, few tiger-range states are routinely performing serum neutralization tests to detect CDV-neutralizing antibodies. This is despite much, if not all, of the necessary equipment being in place in most countries. To facilitate sustainability and future surveillance, we chose not to export the samples but instead established the assay in Nepal (details of this process are given in the Supplementary Material). The minimum facilities required are a reliable power supply, a cell culture lab, an inverted microscope with fluorescence, and a −80 °C freezer. The substitution of GFP in place of luciferase negates the need for an expensive luminometer [30]. Because the pseudotype used in the assay is based on a replication deficient vesicular stomatitis virus, a laboratory rated at biosafety level two or higher is sufficient. Furthermore, all components with the exception of the HEK293DogSLAM cell line can be sourced commercially, but this can be generated by transfecting commercially available HEK293 cells with pDisplay-dogSLAM using linear polyethylenimine [37].

The establishment of national testing facilities that can support coordinated research and surveillance efforts is both highly desirable and achievable and could lead to increased sampling and collaboration. This is relevant when working with large carnivores and threatened species, as the number of animals sampled by a single group or organization is likely to be relatively low [14]. Small sample sizes can frustrate analyses, as highly suggestive results often fail to achieve statistical significance [35]. In Nepal, the existing facilities at the Agricultural and Forestry University, in Rampur and the Biodiversity Conservation Centre in Sauraha provide a suitable base for this, although further development of the molecular lab in Sauraha and collaborations with other institutions such as the National Academy of Sciences and Technology should be encouraged. Cooperation between the DNPWC, the Department of Forests and Soil Conservation, NGOs and universities is already strong, but these efforts could be further augmented through the involvement of local communities, regional veterinary staff, and government veterinary departments. As ever, sample collection remains a key priority. Care should be taken to ensure those working in the field are adequately equipped to collect and store samples correctly.

Whilst further research and surveillance is needed, there are practical steps which can be taken now to try and mitigate the risk of CDV. Dogs may be implicated in transmission, but vaccination of the population would be a significant undertaking and may be unrewarding [19]. Instead, wildlife managers should focus on building resilience. Small and isolated populations are most at risk [11], and efforts should be targeted there. With just 36 (CI: 31–49) tigers in 2022, Shuklaphanta National Park supports one of the smallest tiger populations in Nepal [41]. There is little evidence of immigration [82], and the population could struggle to recover if it suffered substantial losses [11]. Improved connectivity with other populations, including those in India’s western Terai Arc Landscape, would help to address this [82].

Those working with leopards face a different challenge. The species is well connected; however, it is not subject to the same levels of protection or monitoring as the tiger [83]. As such, the impact of any threat, including disease, is hard to quantify and could go undetected until it is too late. Baseline population estimates and commitments to ongoing monitoring are critical to inform the future conservation of the species [83]. As with existing tiger surveys, these efforts would need to be led by local government with the support of conservation NGOs.

## Figures and Tables

**Table 1:** Titers and clinical findings for tigers and leopards that tested positive for neutralizing antibodies to canine distemper virus. Bold font indicates animals that were clinically unwell.

ID	Year	Titer	Healthy	Underweight	Ocular Signs	Respiratory Signs	Gastro-Intestinal Signs	Thickened Pads	Neurological Signs	Mortality	Recovery
**Pt 12**	**2016**	**32**		***✓***			***✓***		***✓***	***✓***	
Pt 23	2019	128	*✓*								
Pt26	2021	91	*✓*								
**Pp5**	**2017**	**362**		***✓***		***✓***	***✓***		***✓***	***✓***	
**Pp7**	**2019**	**23,168**		***✓***			***✓***				***✓***
Pp8	2019	23	*✓*								
Pp11	2019	181	*✓*								
Pp12	2019	128	*✓*								
**Pp17**	**2020**	**2896**		***✓***	***✓***		***✓***	***✓***	***✓***	***✓***	

## Data Availability

All data are included in the manuscript and Supplementary Material.

## Supplementary Materials

The following supporting information can be downloaded at: https://www.mdpi.com/article/10.3390/pathogens12020203/s1, Table S1: Full details of tiger samples and results including date sampled, sex, SNT result, titer and capture site; Table S2: Full details of leopard samples and results including date sampled, sex, SNT result, titer and capture site; Table S3: Details of leopards that died with clinical signs consistent with canine distemper virus infection between 2020 and 2022; Table S4: Summary of tigers and leopards diet studies conducted in Nepal between 2012 and 2022; Supplementary text: Establishing the serum neutralization test (SNT) in Nepal; Figure S1: Comparison of titers as measured by the newly established assay at the Center for Biotechnology, Agricultural and Forestry University in Nepal and the University of Glasgow.
