# Supplementary material for: Canine Distemper Virus in Tigers (Panthera tigris) and Leopards (P. pardus) in Nepal
Source: Pathogens. 2023 Jan 28;12(2):203. doi: 10.3390/pathogens12020203 (PMC9962338; doi:10.3390/pathogens12020203)
Supplement: Supplementary file 1 [file pathogens-12-00203-s001.zip › pathogens-2161423-supplementary.pdf]

Supplementary Materials to accompany

Canine Distemper Virus in tigers *(Panthera tigris)* and leopards *(P. pardus)* in Nepal

Jessica Bodgener*[1, 2], Amir Sadaula*[3], Parbat Jung Thapa[3], Bhijay Kumar Shrestha[4], Kamal Prasad Gairhe[4], Suraj Subedi[5], Kiran Raj Rijal[3], Purushotam Pandey[6], Janardan Dev Joshi[3], Prakriti Kandel[3], Babu Ram Lamichane[3], Chiranjibi Prasad Pokherel[3], Naresh Subedi[3], Ram Chandra Kandel[4], Himal Luitel[7], Navapon Techakriengkrai[8, 9] and Martin Gilbert[1].

Supplementary Materials

Document includes:

- Table S1: Full details of tiger samples and results including date sampled, sex, SNT result, titre and capture site.
- Table S2: Full details of leopard samples and results including date sampled, sex, SNT result, titre and capture site.
- Table S3: Details of leopards that died with clinical signs consistent with canine distemper virus infection between 2020 and 2022.
- Table S4: Summary of tigers and leopards diet studies conducted in Nepal between 2012 and 2022.
- Supplementary text: Establishing the serum neutralization test (SNT) in Nepal.
- Figure S1: Comparison of titres as measured by the newly established assay at the Agricultural and Forestry University in Nepal and the University of Glasgow.
- References

pathogens

MDPI

Table S1. Full details of tiger samples and results including date sampled, sex, SNT result, titre and capture site. CPC = Chitwan-Parsa Complex, BBC = Bardia-Banke Complex, latitude and longitude. * Indicates location has been estimated. Positive samples have been highlighted in **bold.**

ID	Date	Sex	Result	Titre	Site	Latitude	Longitude
Pt1	21-Jan-11	M	-	<8	CPC	27.576222*	84.494334*
Pt2	01-Nov-11	M	-	<8	CPC	27.575394*	84.114591*
Pt3	02-Feb-12	M	-	<8	CPC	27.555477*	84.691397*
Pt4	25-Apr-12	M	-	<8	CPC	27.656816*	84.257839*
Pt5	09-May-13	F	-	<8	CPC	27.592765*	84.407957*
Pt6	09-May-13	F	-	<8	CPC	27.592765*	84.407957*
Pt7	28-Nov-13	M	-	<8	CPC	27.434037*	84.293999*
Pt8	01-Dec-13	M	-	<8	CPC	27.434037*	84.293999*
Pt9	16-Jun-14	M	-	<8	CPC	27.434037*	84.293999*
Pt10	24-Jul-14	M	-	<8	CPC	27.453631	83.907924
Pt11	27-Sep-15	M	-	<8	CPC	27.553408	84.559685
**Pt12**	**08-Feb-16**	**M**	**+**	**32**	**CPC**	**27.565104***	**84.271012***
Pt13	30-Jan-17	F	-	<8	CPC	27.707939*	84.363063*
Pt14	27-Mar-18	M	-	<8	CPC	27.523741*	84.799153*
Pt15	20-Apr-18	M	-	<8	CPC	27.620407*	84.480890*
Pt16	11-May-18	F	-	<8	CPC	27.522759*	84.820672*
Pt17	19-May-18	F	-	<8	CPC	27.522759*	84.820672*
Pt18	29-May-18	M	-	<8	CPC	27.575205*	84.643378*
Pt19	30-Aug-18	M	-	<8	CPC	27.415786*	84.451919*
Pt20	30-Aug-18	M	-	<8	CPC	27.415786*	84.451919*
Pt21	31-Oct-18	F	-	<8	CPC	27.570807*	84.452326*
**Pt22**	**01-Jun-19**	**F**	**+**	**128**	**CPC**	**27.553291***	**84.334956***
Pt23	05-May-20	F	-	<8	CPC	27.549659*	84.330085*
Pt24	14-Feb-21	M	-	<8	CPC	27.203870*	85.073423*
**Pt25**	**17-Mar-21**	**M**	**+**	**91**	**BBC**	**28.517872***	**81.239880***
Pt26	26-Mar-21	F	-	<8	BBC	28.170290*	81.304601*
Pt27	10-Oct-21	F	-	<8	CPC	27.564071*	84.585325*
Pt28	18-Dec-21	M	-	<8	CPC	27.574242*	84.512551*

Table S2. Full details of leopard samples and results including date sampled, sex, SNT result, titre, capture site. CPC = Chitwan-Parsa Complex, KTWR = Koshi Thappu Wildlife Reserve, latitude and longitude. * indicates location has been estimated. Positive samples have been highlighted in **bold.**

ID	Date	Sex	Result	Titre	Site	Latitude	Longitude
Pp1	15-May-13	M	-	<8	Mid-hills	27.687252	83.478198
Pp2	21-Jan-14	M	-	<8	Mid-hills	28.046132*	84.492974*
Pp3	14-Apr-14	M	-	<8	CPC	27.211319	84.758257
Pp4	07-Jul-14	M	-	<8	Mid-hills	27.976703*	84.605844*
**Pp5**	**18-May-16**	**M**	**+**	**362**	**Mid-hills**	**27.830992***	**83.547082***
Pp6	23-Dec-18	F	-	<8	Mid-hills	28.021860	83.395524
**Pp7**	**17-Feb-19**	**M**	**+**	**23168**	**KTWR**	**26.677883**	**87.077280**
**Pp8**	**17-Jul-19**	**F**	**+**	**23**	**Mid-hills**	**28.265564**	**83.890069**
Pp9	05-Aug-19	F	-	<8	Mid-hills	28.315139*	83.962031*
Pp10	12-Nov-19	F	-	<8	Mid-hills	28.018281*	84.433619*
**Pp11**	**01-Dec-19**	**M**	**+**	**181**	**Mid-hills**	**28.008352***	**84.419776***
**Pp12**	**26-Dec-19**	**F**	**+**	**128**	**Mid-hills**	**28.009610***	**84.427546***
Pp13	05-Feb-20	M	-	<8	Mid-hills	28.139384	84.099323
Pp14	17-Mar-20	M	-	<8	Mid-hills	27.880286*	84.733857*
Pp15	16-Apr-20	M	-	<8	Mid-hills	27.572117	84.517815
Pp16	14-May-20	F	-	<8	Mid-hills	27.867870*	83.351882*
**Pp17**	**26-Jul-20**	**M**	**+**	**2896**	**CPC**	**27.454878**	**85.041731**
Pp18	02-Aug-20	M	-	<8	Mid-hills	28.246752*	83.675777*
Pp19	11-Sep-20	F	-	<8	Mid-hills	28.344951*	83.523839*
Pp20	18-Apr-21	M	-	<8	Mid-hills	27.924807*	84.406043*

Table S3. Details of leopards that died with clinical signs consistent with canine distemper virus infection between 2020 and 2022. Only one* of these was blood sampled and included in the study.

Date	Study ID	Age	Sex	Location
23 March 2020	-	Sub-adult	F	Kavre
17 May 2020	-	Adult	M	Kavre
26 July 2020*	Pp17	Adult	M	Hetauda
22 April 2021	-	Adult	M	Kathmandu
30 January 2022	-	Adult	M	Nuwakot
17 March 2022	-	Adult	M	Pokhara

Table S4. Summary of diet studies conducted on tigers and leopards in Nepal between 2012 and 2022. Two further studies were identified but were not included because they provided insufficient detail on sample size or species breakdown [12, 13]. Status of study area – NP = national park, BZ = buffer zone, CA = conservation area, NPA = non-protected area, N = number of scats assessed, % Freq = Relative frequency of occurrence of dog in hairs in scat (%). * This figure relates to all combines all species in the genus Canis i.e. *C. aureus and C. familiaris.*

	Year	Study Area (S.A.)	S.A. Status	N	% Freq.	Ref.
P. pardus fusca						
	2012	Kaski (ACA)	CA	36	2.22	[1]
	2012	Chitwan	NP/BZ	97	3.09	[2]
	2015	Shuklaphanta	NP	42	8.7 *	[3]
	2019	Lamjung (ACA)	CA	40	7.27	[4]
	2020	Kamdi corridor	NPA	60	9.3	[5]
P. tigirs tigris						
	2012	Chitwan	NP/BZ	229	0	[2]
	2015	Shuklaphanta	NP	194	0	[3]
	2015	Chitwan	NP	77	0	[6]
	2016	Bardia / Khata	NP/BZ/NPA	127	0	[7]
	2017	Chitwan	NP	85	0	[8]
	2017	Shuklaphanta	NP	65	0	[9]
	2018	Bardia / Khata	NP/BZ/NPA	75	0	[10]
	2022	Parsa	NP	63	0	[11]

Supplementary text: Establishing the serum neutralization test (SNT) in Nepal

Components

Components of the SNT test were donated by the University of Glasgow, in the UK, and Chulalongkorn University in Thailand and imported to Nepal. This included two cell lines; HEK293T and HEK293DogSLAM; a G complimented recombinant replication deficient vesicular stomatitis virus in which the glycoprotein G had been deleted and replaced with green fluorescent protein (G*rVSVΔG-GFP); and three plasmids, pMD2.G (a gift from Didier Trono, Addgene plasmid # 12259), which encodes for the missing glycoprotein G (ref, see below), and pVR1012-CDV-H and pVR1012-CDV-F which encode for the CDV haemagglutinin (H) and fusion (F) surface proteins respectively.

To validate the assay, 20 domestic dog samples, with known titres, were imported as positive controls. These titres had been measured by the Veterinary Diagnostic Services centre at the University of Glasgow using their standard SNT protocol that uses live virus of the Bussell strain (a Vero cell adapted clone of the Onderstepoort strain of CDV) and Vero cells [14].

Maintaining cell lines

On arrival, frozen cell lines were thawed and recovered in Dulbecco's Modified Eagle Medium (DMEM) supplemented with 10% foetal bovine serum, 100IU/ml penicillin, 100 g/ml streptomycin and 2 mM glutamine, held at 37 ºC and 5% CO_2. Once cells had recovered, the concentration of foetal bovine serum was reduced to 5% (hereafter this medium will be referred to as D5). The HEK293DogSLAM line was also supplemented with 400 μg/ml G418 (Geneticin®, Life Technologies Ltd.).

Propagating G*rVSVΔG-GFP

To generate a stock of the base virus (G*rVSVΔG-GFP), a T75 flask was seeded with 2 x 10^5 HEK293T cells in 10 ml of D5 (a 10 cm cell culture dish could also be used in place of the flask). Once cells were 90% confluent, a transfection mixture was prepared using 10 μg of pMD2.G with 30 μl of linear MW 25,000 polyethylenimine (PEI) (1 mg/ml) (Polysciences) and 2 ml of serum-free DMEM. This was incubated at room temperature for 20 minutes. The medium was then removed from the flask and the transfection mixture was added in its place. This was incubated for 4 hours at 37 ºC and 5% CO_2. Following this 8 ml of D5 was added and the flask and this was incubated at 37 ºC and 5% CO_2 overnight. The following day 0.1 MOI of the imported G*rVSVΔG-GFP was added, and the flask was again incubated overnight at 37 ºC and 5% CO_2. Virus was harvested the following day by drawing off the supernatant from the flask and filtering through a 45 μm syringe filter. The filtered virus was then aliquoted and stored at -80 ºC until needed.

Prior to using the stock virus to generate the pseudotype, it was necessary to calculate the viral titre ($TCID_{50}$ or 50% tissue culture infectious dose). The following concentrations of the harvested stock virus were prepared in quadruplicate on a 96 well plate using D5. 1:10, 1:100, 1:200 then two-fold serial dilutions up to 1:209,715,200. The last four wells were used

as a control and only contained D5. This was termed the dilution plate. 50 µl from each well was then added to a second 96 well plate that had been seeded the day before with HEK293T cells at a density of 2.5 x 10^4 cells/well in 50 µl of D5. This titration plate was incubated for 48 hours at 37 ºC and 5% CO_2. The titration plate was read by examining under fluorescent microscope, where infected cells appeared green. Any wells with infected cells were considered positive, the Spearman Karber method was then used to determine the $TCID_{50}$/ml.

Generating VSV(CDV) pseudotype from G*rVSVΔG-GFP

Ten T75 flasks were seeded with HEK293T cells as described above (a single flask or dish can be expected to produce enough pseudotype to test seven or eight samples, and an additional flask is needed to produce a bald control). Once the flasks were 90% confluent, transfection mixtures were prepared. For each of the pseudotype flasks, 8 µg of each of the CDV protein plasmids (pVR1012-CDV-F and pVR1012-CDV-H), 30 µl of PEI (1 mg/ml) and 200 µl of serum-free DMEM was added to a 1.5 ml tube and incubated at room temperature for 20 minutes. For the bald control flask, 30 µl of PEI (1 mg/ml) and 200 µl of serum-free DMEM was added to a 1.5 ml tube and incubated it in the same way. The transfection mixtures were then added to the respective flasks and incubated at 37 ºC and 5% CO_2 for three hours. All flasks were superinfected with 0.02 MOI of G*rVSVΔG-GFP and incubated for one hour at 37 ºC and 5% CO_2. The existing media was then removed, and the cells were washed three times with PBS. A fresh 10 ml of D5 was then added to each flask and flasks were then incubated for 72 hours at 37 ºC and 5% CO_2. Supernatants were harvested and filtered using a 45 µm syringe filter before being aliquoted and stored at -80 ºC until use.

Prior to using the pseudotype in the assay, it was necessary to calculate the $TCID_{50}$/ml and the level of background infection. The following concentrations of VSV(CDV) pseudotype and the bald control were prepared in quadruplicate on a 96 well plate using D5. 1:10, 1:100, 1:200 then two-fold serial dilutions up to 1:51,200. The final column was used as a negative control and contained only D5. This was referred to as the dilution plate. Another 96 well plate was then seeded with HEK293DogSLAM cells by adding 50 µl of a 4x10^5 cells/ml in D5 to each well. This was referred to as the titration plate. Fifty µl of solution was transferred from each well on the dilution plate to the corresponding well on the titration plate. The titration plate was then incubated at 37 ºC and 5% CO_2 for 48 hours. The $TCID_{50}$ was then calculated in the same way as before. A minimum titre of 5x10^4 $TCID_{50}$/ml is required for a valid serum neutralisation test.

Validation using domestic dog samples

The 20 domestic dog samples that had been imported as positives were tested using the newly established pseudotype assay as described in the main body of the text. Four-fold serial serum dilutions, ranging from 1:16 to 1:16,384, were prepared in quadruplicate on 96 well plates. Each serum sample occupied a quarter of a plate, meaning 5 plates were used in total. A separate plate contained four wells that acted as a serum free control and just contained D5. Five test plates were then prepared by diluting the VSV(CDV) pseudotype to

$5x10^4$ $TCID_{50}$/ml, 50 µl of this virus solution was then added to each well on a 96 well plate. Again, a separate plate was used for the serum free control, in this case only four wells were filled with virus solution. Fifty µl of serum solution was then transferred from each well on the dilution plates to the corresponding well on the test plates. Test plates were then incubated for one hour at 37 ºC and 5% CO_2. Following this 50 µl of a 4×10^5 cells/ml solution of HEK293DogSLAM was added to each well and the plates were incubated for a further 48 hours. Results were read as described in the main body of the text.

The serum free negative control tested negative. All 20 positive dog samples tested positive. Corresponding titres were plotted against each other (Figure A1.). The correlation coefficient was calculated using Microsoft Excel as 0.82 (CI: 0.57-0.92). Given that different methods were used, this indicated a high level of agreement and served to validate the assay[14].

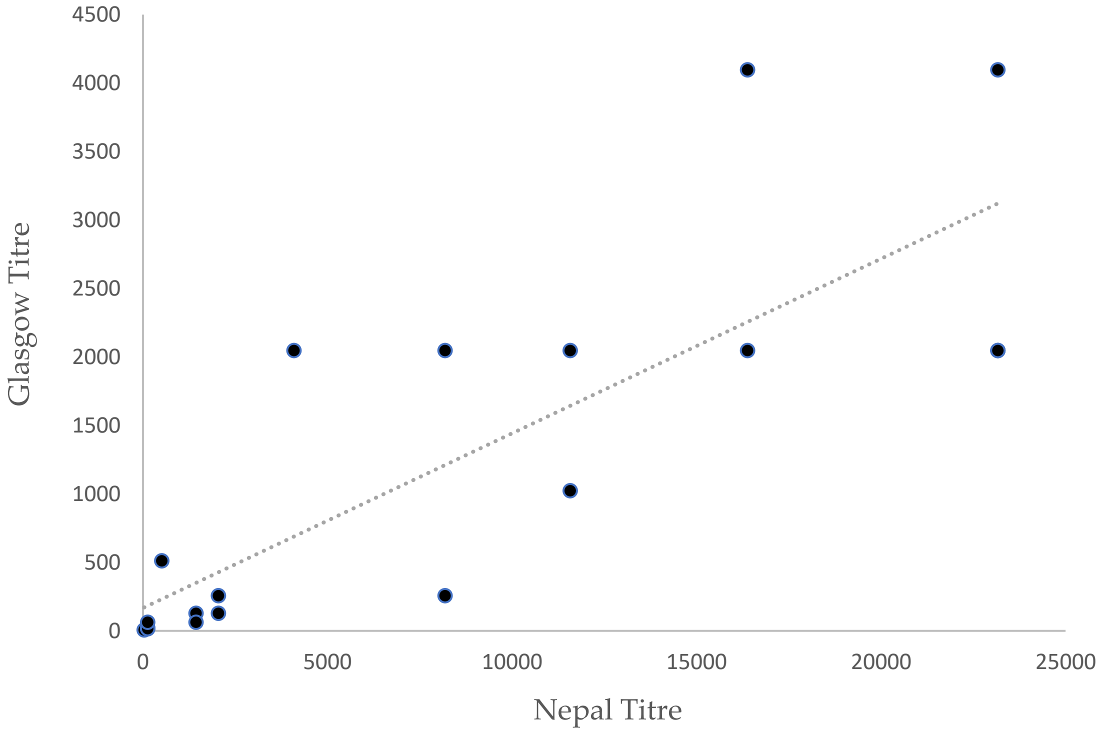

Figure S1. Comparison of titres as measured by the newly established assay at the Agricultural and Forestry University in Nepal and the University of Glasgow. The method of assessment differed slightly with the Nepali assay using a pseudotype based assay and quantitative method of assessment whereas the University of Glasgow used live CDV virus and a qualitative assessment of CPE. The correlation coefficient was calculated as 0.82.

References

1. Koirala, R.; Aryal, A.; Amiot, C.; Adhikari, B.; Karmacharya, D. and Raubenheimer, D. Genetic identification of carnivore scat: Implication of dietary information for human–carnivore conflict in the Annapurna Conservation Area, Nepal. Zool. Ecol. 2012. 22: p. 137-143.
2. Bhattarai, B.P. and Kindlmann, P. Interactions between Bengal tiger (Panthera tigris) and leopard (Panthera pardus): implications for their conservation. *Biodivers. Conserv.* **2012**. 21(8): p. 2075-2094.
3. Lovari, S.; Pokheral, C.P.; Jnawali, S.R.; Fusani,L. and Ferretti, F. Coexistence of the tiger and the common leopard in a prey-rich area: the role of prey partitioning. *J. Zool.* **2015**. 295(2): p. 122-131.
4. Kandel, S., Panthera pardus fusca (Family: Felidae) diet composition from Lamjung, Nepal. *Environ. Ecol. Res.* **2019**. 7(4): p. 253-258
5. Kandel, S.R.; Lamichhane, B.R. and Subedi, N. Leopard (Panthera pardus) density and diet in a forest corridor of Terai: implications for conservation and conflict management. *Wildl. Res.* **2020**. 47(6): p. 460-467.
6. Lamichhane, S. and Jha, B. Prey selection by Bengal Tiger Panthera tigris tigris (Mammalia: Carnivora: Felidae) of Chitwan National Park, Nepal. J. Threat. Taxa. 2015. 7: p. 8081-8088
7. Wegge, P.; Yadav, S.K. and Lamichhane. B.R., Are corridors good for tigers (Panthera tigris) but bad for people? An assessment of the Khata corridor in lowland Nepal. Oryx 2018. 52(1): p. 35-45.
8. Bhandari, S.; Chalise, M.K. and Pokharel, C.P. Diet of Bengal tigers (Panthera tigris tigris) in Chitwan National Park, Nepal. *Eur. J. Ecol.* **2017**. 3(1): p. 80-84.
9. Baniya, R.K.; Baniya, C.B.; Mou, P and Ge, J. Prey selection by tiger (Panthera tigris tigris) in Shuklaphanta Wildlife Reserve Nepal. *Int. J. Sci.* **2017**. 3: p. 90-99.
10. Upadhyaya, S.K., Musters, C.J.M.; Lamichhane, B.R.; de Snoo, G.R.; Thapa, P.; Dhakal, M.; Karmacharya, D.; Shrestha, P.M. and de Iongh, H.H. An insight into the diet and prey preference of tigers in Bardia National Park, Nepal. *Trop. Conserv. Sci.* **2018**. 11: p. 1-9
11. Lamichhane, S.; Thanet, D.R.; Regmi, P.R.; Maharjan, A. and Lamichhane, B.R. Dietary composition and prey preference of Royal Bengal Tiger (Panthera tigris tigris, Linnaeus 1758) of Parsa National Park, Nepal. *Eur. J. Ecol.* **2022**. **8**(1): p. 34-38
12. Lamichhane, B.R.; Leirs, H.; Persoon, G.A.; Subedi, N.; Dhakal, M.; Oli, B.N.; Reynaert, S.; Sluydts, V.; Pokheral, C.P.; Poudyal, L.P. et al Factors associated with co-occurrence of large carnivores in a human-dominated landscape. *Biodivers. Conserv*. **2019**. 28: p. 1473–1491
13. Bhattarai, B. and Kindlmann P. Human Disturbance is the Major Determinant of the Habitat and Prey Preference of the Bengal Tiger (Panthera tigris tigris) in the Chitwan National Park, Nepal. Eur. J. Ecol. 2018. 4: p. 13-21.
14. Logan, N.; McMonagle, E.; Drew, A.A.; Takahashi, E.; McDonald, M.; Baron, M.D.; Gilbert, M.; Cleaveland, S.; Haydon, D.T.; Hosie, M.J. et al Efficient generation of

vesicular stomatitis virus (VSV)-pseudotypes bearing morbilliviral glycoproteins and their use in quantifying virus neutralising antibodies. *Vaccine* **2016**. 34(6): p. 814-22
